# Synthesis and Antitubercular Activity of Heteroaromatic Isonicotinoyl and 7-Chloro-4-Quinolinyl Hydrazone Derivatives

**DOI:** 10.1100/tsw.2010.124

**Published:** 2010-07-07

**Authors:** Marcelle de L. Ferreira, Raoni S.B. Gonçalves, Laura N. de F. Cardoso, Carlos R. Kaiser, Andre L.P. Candéa, Maria das Graças M. de O. Henriques, Maria C.S. Lourenço, Flávio A.F.M. Bezerra, Marcus V. N. de Souza

**Affiliations:** ^1^ FioCruz-Fundação Oswaldo Cruz, Instituto de Tecnologia em Fármacos-Far Manguinhos, Manguinhos, Rio de Janeiro, Brazil; ^2^ Departamento de Química Orgânica, Instituto de Química, Universidade Federal do Rio de Janeiro, Rio de Janeiro, Brazil; ^3^ Instituto de Pesquisas Clínica Evandro Chagas – IPEC, Manguinhos, Rio de Janeiro, Brazil

**Keywords:** tuberculosis, quinoline, isoniazid, drugs

## Abstract

Two series of *N’*(E)-heteroaromatic-isonicotinohydrazide derivatives (3a-f and 4a-b) and 1-(7-chloroquinolin-4-yl)-2-[(heteroaromatic)methylene]hydrazone derivatives (5a-f and 6a-b) have been synthesized and evaluated for their *in vitro* antibacterial activity against *Mycobacterium tuberculosis* H_37_Rv. Several compounds were noncytotoxic and exhibited significant minimum inhibitory concentration (MIC) activity (3.12, 2.50, 1.25, or 0.60 μg/mL), which can be compared to that of the first-line drugs ethambutol (3.12 μg/mL) and rifampicin (2.0 μg/ml). These results can be considered an important starting point for the rational design of new leads for anti-TB compounds.

### INTRODUCTION

Tuberculosis (TB) is the most important infectious cause of death worldwide. According to the World Health Organization (WHO), more than 2 billion people are infected with TB bacilli (*Mycobacterium tuberculosis*) and a total of 1.77 million people died from TB in 2007[1]. The lack of new anti-TB drugs, the coinfection with HIV/AIDS, and the advent of resistant strains to the current therapy are the main causes responsible for TB resurgence[2]. Among these problems, the emergence of drug-resistant TB is especially alarming. According to the WHO, 511,000 cases of multidrug-resistant TB (MDR-TB), strains resistant to isoniazid and rifampicin, occurred in 2007 (4.9% of all cases). Among these cases, 289,000 were new cases and 221,000 were cases that had been previously treated for TB. Another important factor in TB treatment worldwide is the advent of extensively drug-resistant TB (XDR-TB), which is commonly defined as MDR-TB plus resistance to any fluorquinolone and to, at least, one of the three injectable second-line anti-TB drugs used in TB treatment (capreomycin, kanamycin, and amikacin)[1,3]. By the end of 2008, 55 countries and territories had reported at least one case of XDR-TB. The WHO estimates that 19% of MDR cases are in fact XDR-TB and the cure is possible for up to 50–60% of the people affected[1].

Due to the high impact of MDR and XDR in TB treatment, there is an urgent need for new drugs to treat this disease efficiently. In this context, isoniazid (INH) derivatives have been found to possess potential anti-TB activities[4,5,6]. INH is one of the most powerful synthetic agents against the *M. tuberculosis* complex and it has an important bactericidal activity against the replicating bacteria. Moreover, INH is a prodrug, which needs a previous *in vivo* activation to exercise its anti-TB activity. The enzyme responsible for this function is called KatG. After INH activation, an isonicotinoyl radical is produced**,** which reacts with the nicotinamide group of NAD (nicotinamide adenine dinucleotide) to yield the INH-NAD adduct. This adduct mainly inhibits and binds to *trans*-2-enoyl-ACP reductase, encoded by the *InhA* gene, which promotes the elongation phase of the FAS-II (fatty acid synthetase II) system. The inhibition of this enzyme interrupts the mycolic biosynthesis leading to cell lysis[7].

Due to the significance of this drug for TB treatment, the advent of INH-resistant strains is very alarming. The majority of INH-resistant strains demonstrate deletion or point mutations in the *M. tuberculosis* katG gene, which is responsible for INH activation[8]. Moreover, it is probable that $Mn^{3+}$ ions could facilitate the formation of isonicotinic acyl radicals and KatG participates in isoniazid activation by increasing the rate of the conversion of $Mn^{2+}$ to $Mn^{3+}$ ions. Due to the ability of hydrazone derivatives in metal chelation[9] and generation of metal ion–induced radical intermediates[10,11,12], we decided to investigate the potential anti-TB activity of a series of heteroaromatic hydrazones derived from INH (**3a-f** and **4a-b,** see Scheme 1). Another aim of this article is to compare the biological activity of the INH derivatives to a series of heteroaromatic 7-chloro-4-quinolinylhydrazones (**5a-f** and **6a-b,** see Scheme 1). Recently, we reported the synthesis and anti-TB activity of a series of monosubstituted 7-chloro-4-quinolinylhydrazones, which demonstrated relevant minimum inhibitory concentration (MIC) between 12.5 and 2.5 μg/mL[13]. Hence, this report is also very important in order to continue the study of the structure-activity relationship of this class of compounds.

The criteria used to select the five-member heterocyclic nuclei was based on isosteric replacements: (1) substitution of the oxygen atom of the furane ring (**1a**) by sulfur (**1d**) or nitrogen (**1e**) and (2) substitution of –CH= by –N= in the pyrrole ring **(1e)** to give an imidazole ring (**1f**); whereas the six-member heterocyclic (**2a-b**) was chosen in order to analyze the influence of the introduction of the nitrogen atom in the phenyl ring on the biological activity of this series.

## EXPERIMENTAL PROCEDURES

### General Procedures

Melting points were determined on a Buchi apparatus and are uncorrected. Infrared spectra were recorded in a Thermo Nicolet Nexus 670 spectrometer, as potassium bromide pellets and frequencies are expressed in $cm^{-1}$. Mass spectra (ESI assay in solution of ammonium chloride) were recorded in Micromass ZQ Waters mass spectrometer. NMR spectra were recorded in a Bruker Avance 400 operating at 400.00 MHz ($^{1}H$) and 100.0 MHz ($^{13}C$), and Bruker Avance 500 spectrometer operating at 500.00 MHz ($^{1}H$) and 125.0 MHz ($^{13}C$), in deuterated dimethylsulfoxide. Chemical shifts are reported in ppm (δ) relative to tetramethylsilane and *J*-coupling in Hertz (Hz). Proton and carbon spectra were typically obtained at room temperature. TLC plates, coated with silica gel, were run in a chloroform/methanol (9:1) mixture and spots were developed in ultraviolet and solution of ninhidrine (0.2% p/v in ethanol).

#### *General Procedures for Synthesis of N´-(E)-Heteroaromatic-Isonicotinohydrazide Derivatives (3a-f and 4a-b)*

The synthesis of *N´*-(*E*)-heteroaromatic-isonicotinohydrazide derivatives (**3a-f** and **4a-b**) was prepared by the reaction between isoniazid (1.0 equiv.) and the appropriate heteroaromatic aldehyde (**1a-f** and **2a-b**) (1.2 equiv.) in a mixture of ethanol and water distillate (1:1, 10 mL); initially, dissolving isoniazid in water distillate (5 mL) and adding the respective heteroaromatic aldehyde in ethanol (5 mL). After stirring for 4–24 h at room temperature, the resulting mixture was concentrated under reduced pressure and the residue purified by washing with cold $Et_2O$ (3 × 10 mL), leading to the pure derivatives (**3a-f** and **4a-b**) as a solid in 56–91% yields.

#### N'-[(E)-(1H-Imidazol-2-yl)Methylidene]Isonicotinohydrazide (3f)

**Yield**: 56%; **mp**: 198–200°C; **$^1$H NMR[500 MHz (FIDRES ± 0.15 Hz), DMSO-*d*$_6$] δ:** 13.38 (1H; br; NH); 12.08 (1H; br; NH); 8.80 (2H; d; *J* = 5.5 Hz; $H_2$ and $H_6$); 8.37 (1H; s; N=C-H); 7.84 (2H; br; $H_3$ and $H_5$); 7.69 (2H; m; $H_{7'}$ and $H_{8'}$). **$^{13}$C NMR (125 MHz, DMSO-*d*$_6$) δ:** 160.9; 150.2; 141.8; 140.7; 136.6; 122.3; 121.4 ppm. **IV$\nu_{max}$ (cm$^{-1}$; KBr pellets):** 3233 (N-H); 1655 (C=O). **MS/ESI:** *m/z*$[M-H]^{+\cdot}$: 214.

### *General Procedures for Synthesis of 7-Chloro-4-Quinolinylhydrazone Derivatives (5a-f and 6a-b)[13]*

The 7-chloro-4-quinolinylhydrazone derivatives (**5a-f** and **6a-b**) were obtained by the reaction between 7-chloro-4-hydrazinoquinoline (1.03 mmols) and the appropriate heteroaromatic aldehyde (**1a-f** and **2a-b**) (1.24 mmols) in ethanol (5 mL). After stirring for 3–30 h at room temperature, the resulting mixture was concentrated under reduced pressure and the residue purified by washing with cold $Et_2O$ (3 × 10 mL), leading to the pure derivatives (**5a-f** and **6a-b**) as solids in 58–92% yields.

#### 1-(7-Chloroquinolin-4-yl)-2-[(5-Nitro-Furan-2-yl)Methylene]Hydrazine (5a)

**Yield**: 85%; **mp**: 238–240°C; **$^1$H NMR[500 MHz (FIDRES ± 0.15 Hz), DMSO-*d*$_6$] δ:** 11.70 (1H; br; NH), 8.35 (2H; m; $H_2$ and $H_5$), 8.30 (1H; s; N=C-H), 7.81 (2H; d; *J* = 3.7 Hz; $H_{7'}$ and $H_6$), 7.55 (1H; br; $H_8$); 7.22–7.26 (2H; m; $H_3$ and $H_{8'}$). **IV$\nu_{max}$ (cm$^{-1}$; KBr pellets):** 3158 (N-H); 1571 (C=N). **MS/ESI:** *m/z*$[M-H]^{+\cdot}$: 315.

#### 1-(7-Chloroquinolin-4-yl)-2-[(5-Nitro-Thiophen-2-yl)Methylene]Hydrazine (5c)

**Yield**: 92%; **mp**: 189–190°C; **$^1$H NMR[500 MHz (FIDRES ± 0.15 Hz), DMSO-*d*$_6$] δ:** 11.91 (1H; br; NH), 8.55 (2H; m; $H_2$ and $H_5$), 8.22 (1H; s; N=C-H), 8.08 (1H; d; *J* = 4.4 Hz; $H_{7'}$); 7.53 (1H; d; *J* = 1.9 Hz; $H_8$); 7.48–7.51 (2H; m; $H_{8'}$ and $H_6$); 6.98 (1H; d; *J* = 7.4 Hz; $H_3$). **IV$\nu_{max}$ (cm$^{-1}$; KBr pellets):** 3182 (NH); 1585 (C=N). **MS/ESI:** *m/z*$[M+H]^{+\cdot}$: 333.

#### 1-(7-Chloroquinolin-4-yl)-2-[(2-Pyridyl)Methylene]Hydrazine (6a)

**Yield**: 61%; **mp**: 210–211°C; **$^1$H NMR[500 MHz (FIDRES ± 0.15 Hz), DMSO-*d*$_6$] δ:** 11.46 (br; 1H; NH), 8.61 (2H; m; $H_2$ and $H_5$); 8.41 (1H; s; N=C-H), 8.38 (1H; d; *J* = 7.9 Hz; $H_{6'}$or $H_{9'}$), 8.09 (1H; d; *J* = 7.9 Hz; $H_{6'}$or $H_{9'}$), 7.85–7.88 (2H; m; $H_8$ and $H_{7'}$ or $H_{8'}$), 7.56 (1H; br; $H_6$), 7.37–7.45 (2H; m; $H_3$ and $H_{7'}$ or $H_{8'}$). **IV$\nu_{max}$ (cm$^{-1}$; KBr pellets):** 3419 (N-H); 1442 (C=N). **MS/ESI:** *m/z*$[M-H]^{+\cdot}$: 281.

### General Procedures for Biological Tests

#### Antimycobacterial Activity

Briefly, 200 μL of sterile deionized water was added to all outer-perimeter wells of sterile 96 well plates (falcon, 3072: Becton Dickinson, Lincoln Park, NJ) to minimize evaporation of the medium in the test wells during incubation. The 96 plates received 100 μL of the Middlebrook 7H9 broth (Difco Laboratories, Detroit, MI) and a serial dilution of the compounds (**3a-f, 4a-b, 5a-f,** and **6a-b**) was made directly on the plate. The final drug concentration tests were 0.01–100 μg/mL. Plates were covered and sealed with parafilm and incubated at 37ºC for 5 days. After this time, 25 μL of a freshly prepared 1:1 mixture of Alamar Blue (Accumed International, Westlake, OH) reagent and 10% Tween 80 was added to the plate and incubated for 24 h. A blue color in the well was interpreted as no bacterial growth and a pink color was scored as growth. The MIC was defined as the lowest drug concentration, which prevented a color change from blue to pink.

#### Cell Viability Assay

The cellular viability for a macrophage cell line J774 (ATCC TIB-67™) was determined by Mosman's MTT (3-(4,5-dimethylthylthiazol-2yl)-2,5-dimethyl tetrazolium bromide; Merck) microcultured tetrazolium assay. We evaluated macrophages in the presence and absence of test compounds (**3a-c, 3e-f, 4a-b, 5a-f,** and **6a**). The cells were plated in flat-bottom 96 well plates ($2.5 \times 10^6$ cells/well/100 μL) cultured for 24 h in a controlled atmosphere ($CO_2$ 5% at 37°C), and nonadherent cells were washed by gentle flushing with RPMI 1640 supplemented with fetal bovine serum (10%) and gentamicin (25 μg/mL). Adherent cells were infected or not with BCG ($2.5 \times 10^6$ UFC/well/100 μL) cultured in the presence of medium alone, Tween 20 (3%) (live and dead controls, respectively), or different concentrations of compounds (1.0, 10.0, and 100 μg/mL) in a triplicate assay. After 48 h, stock MTT solution (5 mg/mL of saline; 20 mL/well) was added to the culture and 4 h later, the plate was centrifuged for 2 min at 2800 rpm, supernatant was discharged, and dimethyl sulfoxide (DMSO) (100 μL/well) was added to formazan crystals solubilization, and the absorbance was ready at 540 nm in a plate reader (Biorad – 450).

### RESULTS AND DISCUSSION

### Chemistry

The synthetic routes for the preparation of the *N´*-(*E*)-heteroaromatic-isonicotinohydrazide derivatives (**3a-f** and **4a**) and the heteroaromatic 7-chloro-4-quinolinylhydrazone derivatives (**5a-f** and **6a-b**) are summarized in Scheme 1. Basically, these compounds were obtained from reactions of isoniazid or 7-chloro-4-hydrazinoquinoline and heteroaromatic aldehydes in EtOH: $H_2O$ (1:1) or EtOH at room temperature, respectively (Scheme 1 and Table 1).

All the compounds were identified by the spectral data. In general, IR spectra of INH derivatives (**3a-f** and **4a-b**) showed the C=O peak at 1648–1678 $cm^{-1}$ and the NH stretching vibrations at 3015–3270 $cm^{-1}$. The nuclear magnetic resonance spectra ($^1H$ NMR) showed the hydrazide (NH) proton as a singlet at 12.46–11.78 ppm and the imine proton (N=C-H) at 8.78–8.37 ppm. The $^{13}C$ NMR spectrum showed the C=O signals at 161.9–161.4 ppm and C=N signals at 146.1–140.1 ppm. For the quinoline derivatives (**5a-f** and **6a-b**), the IR spectra showed the N=C stretching vibration at 1612–1576 $cm^{-1}$. Specifically, in the $^1H$ NMR spectra, the imine proton (N=C–$\underline{H}$) appears as a singlet in the range 8.81–8.29 ppm.

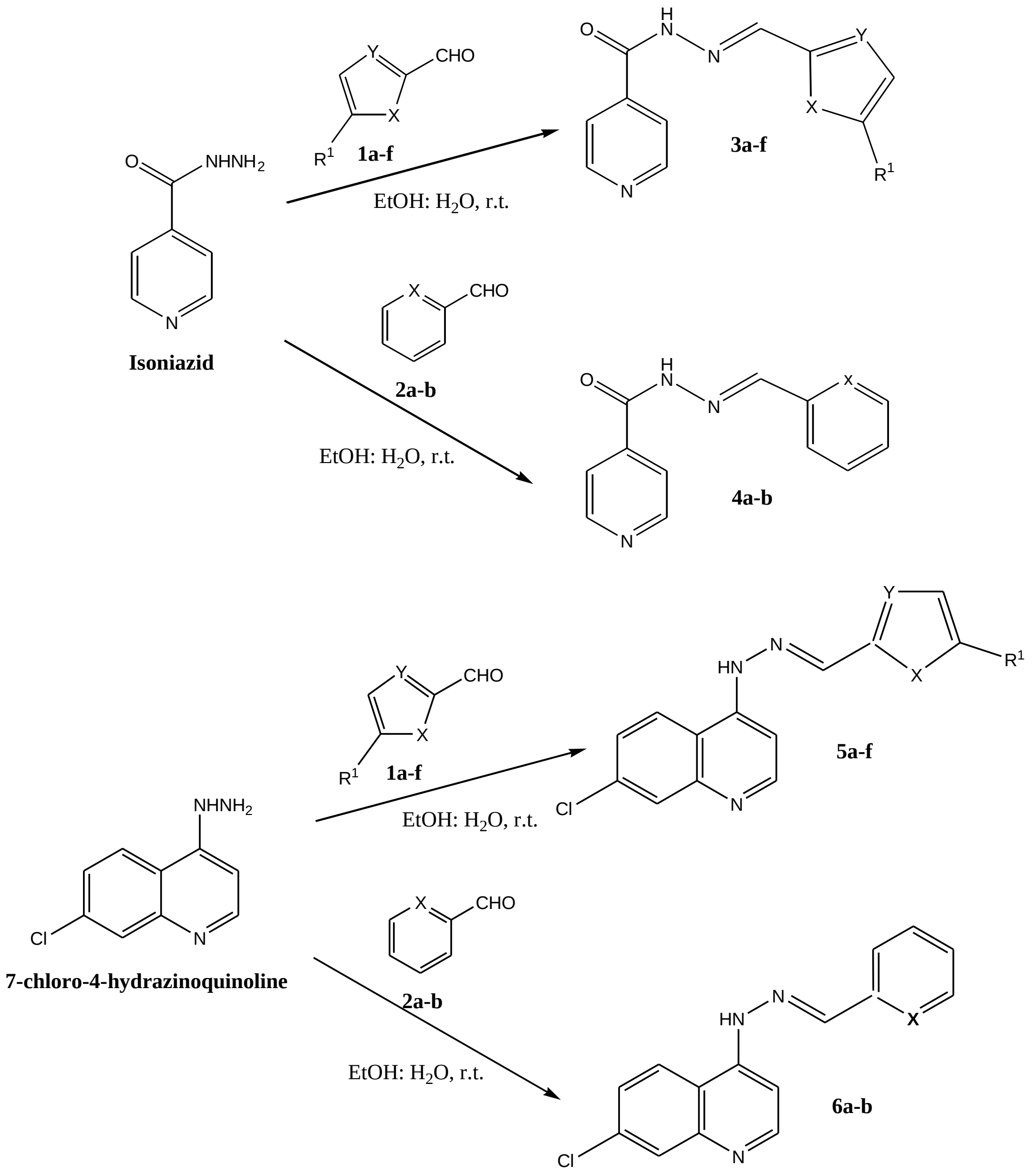

| Entry | X | Y | $R_1$ |
|---|---|---|---|
| **1a** | O | CH | $NO_2$ |
| **1b** | O | CH | H |
| **1c** | S | CH | $NO_2$ |
| **1d** | S | CH | H |
| **1e** | NH | CH | H |
| **1f** | NH | N | H |
| **2a** | N | --- | --- |
| **2b** | CH | --- | --- |

**SCHEME 1**. Synthetic routes for the preparation of the *N´*-(*E*)-heteroaromatic-isonicotinohydrazide derivatives (**3a-f** and **4a-b**) and the heteroaromatic 7-chloro-4-quinolinylhydrazone derivatives (**5a-f** and **6a-b**).

**TABLE 1**
**Yields and Melting Points of Isonicotinoyl Hydrazones and 7-Chloro-4-Quinolinylhydrazones (3a-f, 4a-b, 5a-f, and 6a-b)**

| Entry | Yield (%) | m.p. (°C) |
|---|---|---|
| 3a | 75 | 184–186[14] |
| 3b | 82 | 179[15] |
| 3c | 90 | 188–189[14] |
| 3d | 87 | 224–226[15] |
| 3e | 91 | 234–235[16] |
| 3f | 56 | 198-200 |
| 4a | 79 | 122[15] |
| 4b | 83 | 160–161[17] |
| 5a | 85 | 238-240 |
| 5b | 78 | 210–212[18] |
| 5c | 92 | 189–190 |
| 5d | 83 | 231–232[18] |
| 5e | 82 | 219–220[18] |
| 5f | 58 | 274–275[18] |
| 6a | 61 | 210–211 |
| 6b | 70 | 223–225[13] |

### Antimycobacterial Activity

The antimycobacterial activities of the derivatives **3a-f**, **4a-b**, **5a-f,** and **6a-b** were assessed against *M. tuberculosis* ATCC 27294[19] using the microplate Alamar Blue assay (MABA)[20] (Table 2). This nontoxic methodology uses a thermally stable reagent, and shows good correlation with proportional and BACTEC radiometric methods[21,22].

When these two different series of compounds were compared, it was observed that among the compounds with five-member heterocyclic nucleus (**3a-f** vs. **5a-f**), the quinoline derivatives are more active than INH derivatives, except in the case of **3e** and **5e**. However, the comparison between the six-member compounds (**4a-b** vs. **6a-b**) showed that INH derivatives were more active than quinoline derivatives. These data might indicate that biological activity of quinoline derivatives is more susceptible to bulk effects than INH derivatives. This hypothesis can be more detailed if we compare the five-member heterocyclic nucleus [**5d** (S), **5b** (O), **5e** (NH), and **5f** (N plus NH)] bounded to quinoline derivatives. It was observed that there is no difference in the biological activity of these compounds (all derivatives showed MIC = 3.12 µg/mL), but with the increase of size ring (six-member compounds, **6a** and **6b**), the biological activity decreases four times in the case of **6a** or completely disappears in the case of **6b**.

Furthermore, when the compounds are compared into the same series (**3a** vs. **3b**, **5a** vs. **5b,** and **5c** vs. **5d**), it was observed that all the nitro derivatives (**3a**, **5a,** and **5c**) were more active than the other compounds (**3b**, **5b,** and **5d**), suggesting that the nitro group is an important feature to modulation of biological activity in these series.

Moreover, when the derivatives **4a** and **4b**, **6a** and **6b** are compared, it was observed that the compounds without the nitrogen atom were less active, suggesting that the presence of this atom in the six-member compounds also seems to be important for the biological activity in both series.

**TABLE 2**
**Antimycobacterial Activities and *c*logP Measurements of INH and 7-Chloro-4-Quinolinylhydrazone Derivatives (3a-f, 4a-b, 5a-f, and 6a-b)**

| Entry | MIC[a] (µg/mL) | *c*Log*P*[b] | Entry | MIC[a] (µg/mL) | *c*Log*P*[b] |
|---|---|---|---|---|---|
| 3a | 3.12 | 1.07 | 5a | 2.50 | 4.99 |
| 3b | 25 | 1.15 | 5b | 3.12 | 4.91 |
| 3c | 1.25 | 1.71 | 5c | 1.25 | 5.63 |
| 3d | N.D.[c] | 1.79 | 5d | 3.12 | 5.52 |
| 3e | 1.25 | 0.96 | 5e | 3.12 | 4.81 |
| 3f | 25 | –0.10 | 5f | 3.12 | 3.75 |
| 4a | 0.60 | 1.81 | 6a | 12.5 | 4.36 |
| 4b | 3.12 | 0.64 | 6b | Resistant | 5.65 |
| Isoniazid | 0.2 | –0.97 | Isoniazid | 0.2 | –0.97 |

[a] Minimum inhibitory concentration.

[b] Calculated using www.molinspiration.com.

[c] N.D. = not determined due to the occurrence of color interference during the assay.

### Cell Viability Assay

All the active compounds (**3a-c**, **3e-f**, **4a-b**, **5a-f,** and **6a**) were selected for evaluation of their cytotoxicities by Mosman's assay. The cellular viability in the presence and absence of the test compounds (**3a-c**, **3e-f**, **4a-b**, **5a-f,** and **6a**) was determined by Mosman's MTT (3-(4.5-demethylthylthiazol-2-yl)-2.5-dimethyltetrazolium bromide; Merck) microcultured tetrazolium assay[23,24]. The results were represented as percentage cell viability (Table 3).

**TABLE 3**
**Data of the Cellular Viability for a Macrophage Cell Line J774 (ATCC TIB-67™) by Mosman's Assay**

| Entry | % Cell Viability/Dose (µg/ml) | | | Entry | % Cell Viability/Dose (µg/ml) | | |
|---|---|---|---|---|---|---|---|
| | **1** | **10** | **100** | | **1** | **10** | **100** |
| 3a | 91 | 80 | 95 | 5a | 90 | 100 | 17 |
| 3b | 91 | 90 | 87 | 5b | 78 | 65 | 10 |
| 3c | 95 | 80 | 45 | 5c | 97 | 87 | 36 |
| 3d | — | — | — | 5d | 95 | 89 | 28 |
| 3e | 92 | 95 | 89 | 5e | 86 | 25 | 13 |
| 3f | 80 | 76 | 58 | 5f | 82 | 84 | 15 |
| 4a | 96 | 100 | 53 | 6a | 100 | 27 | 13 |
| 4b | 95 | 98 | 74 | 6b | — | — | — |
| Isoniazid | 97 | 100 | 100 | Isoniazid | 97 | 100 | 100 |

This table shows that the compounds **3a-c, 3e, 4a-b, 5a, 5c-d,** and **6a** did not kill more than 10% of the host cells in the minimum concentration tested. In general, INH derivatives were less cytotoxic than quinoline derivatives. Another important observation is that the presence of the nitro group in these compounds did not lead to the increase of their cytotoxicities (see **3a** vs. **3b**, **5a** vs. **5b,** and **5c** vs. **5d**).

### CONCLUSION

The synthesis of 16 INH and 7-chloro-4-hydrazinoquinoline heteroaromatic hydrazone derivatives (**3a-f**, **4a-b**, **5a-f,** and **6a-b**) was performed in good yields (56–92%). Among them, four are new compounds (**3f**, **5a**, **5c,** and **6a**). All these compounds were submitted to antimycobacterial activity evaluation and 14 derivatives (**3a-c**, **3e-f**, **4a-b**, **5a-f,** and **6a**) exhibited MIC values between 25 and 0.60 µg/mL. Therefore, these compounds were selected for the evaluation of their cytotoxicities by Mosman's assay. Among these derivatives, **3a-c, 3e, 4a, 5a, 5c-d,** and **6a** were not cytotoxic to host cells in the effective concentrations to inhibit the growth of *M. tuberculosis*. Furthermore, the compounds **3a, 3c, 3e, 4a, 5a, 5c,** and **5d** exhibited a significant activity (3.12, 2.50, 1.25, or 0.60 µg/mL) when compared to the first-line drugs, such as ethambutol (MIC = 3.12 µg/mL) and rifampicin (2.0 µg/ml), and could be considered a good starting point to find new lead compounds in the fight against TB.
